# Crystal structure of *catena*-poly[[[bis­(pyridine-4-carbo­thio­amide-κ*N* ^1^)cadmium]-di-μ-thio­cyanato-κ^2^ *N*:*S*;κ^2^ *S*:*N*] methanol disolvate]

**DOI:** 10.1107/S2056989016002632

**Published:** 2016-02-20

**Authors:** Tristan Neumann, Inke Jess, Christian Näther

**Affiliations:** a Institut für Anorganische Chemie, Christian-Albrechts-Universität zu Kiel, Otto-Hahn-Platz 6, D-24118 Kiel, Germany

**Keywords:** crystal structure, cadmium thio­cyanate, coordination polymer, pyridine-4-carbo­thio­amide, hydrogen bonding

## Abstract

The asymmetric unit of the polymeric title compound, {[Cd(NCS)_2_(C_6_H_6_N_2_S)]·2CH_3_OH}_*n*_, consists of one cadmium(II) cation that is located on a centre of inversion as well as one thio­cyanate anion, one pyridine-4-carbo­thio­amide ligand and one methanol mol­ecule in general positions. The Cd^II^ cations are octa­hedrally coordinated by the pyridine N atom of two pyridine-4-carbo­thio­amide ligands and by the S and N atoms of four thio­cyanate anions and are linked into chains along [010] by pairs of anionic ligands. These chains are further linked into layers extending along (201) by inter­molecular N—H⋯O and O—H⋯S hydrogen bonds. One of the amino H atoms of the pyridine-4-carbo­thio­amide ligand is hydrogen-bonded to the O atom of a methanol mol­ecule, and a symmetry-related methanol mol­ecule is the donor group to the S atom of another pyridine-4-carbo­thio­amide ligand whereby each of the pyridine-4-carbo­thio­amide ligands forms two pairs of centrosymmetric N—H⋯S and O—H⋯S hydrogen bonds. The methanol mol­ecules are equally disordered over two orientations.

## Abstract 2

The crystal structure of the title compound consists of Cd^II^ cations that are linked by thio­cyanato anions into chains which are further connected into layers by inter­molecular N—H⋯O and O—H⋯S hydrogen bonding *via* additional methanol mol­ecules.

## Chemical context   

Thio­cyanato anions are versatile ligands that can coordinate to metal cations in different ways (Näther *et al.*, 2013 ▸). In this context, compounds in which paramagnetic metal cations are linked into chains by μ-1,3 bridging anionic ligands are of special inter­est, because they can show different magnetic behavior (Palion-Gazda *et al.*, 2015 ▸). This is the case *e.g.* for compounds of general composition *M*(NCS)_2_(*L*)_2_ (*M* = Mn, Fe, Co and Ni; *L* = pyridine derivative) that frequently show cooperative magnetic properties like ferromagnetic or anti­ferromagnetic ordering or a slow relaxation of the magnetization indicative for single chain magnetic behavior (Näther *et al.*, 2013 ▸; Wöhlert *et al.*, 2011 ▸; Boeckmann & Näther, 2012 ▸; Werner *et al.*, 2015**a* ▸,b* ▸). Unfortunately, compounds with a bridging coordination are frequently less stable than those in which these anionic ligands are only *N*-terminally coordinating. Hence, we have developed an alternative synthesis procedure which is based on thermal decomposition of suitable precursor compounds and leads directly to the formation of the desired compounds (Näther *et al.*, 2013 ▸). However, following this procedure only microcrystalline materials are obtained. This is the reason why we are also inter­ested in the diamagnetic cadmium analogues. This metal cation is much more chalcophilic than most paramagnetic cations, which means that the desired compounds with a bridging coordination of the anionic ligands can easily be crystallized and characterized by single crystal X-ray diffraction (Wöhlert *et al.*, 2013 ▸). In several cases, the resulting structures are isotypic to the paramagnetic analogues and therefore the latter can be refined by the Rietveld method using the crystallographic data of the respective Cd^II^ compound (Wöhlert *et al.*, 2013 ▸). In the scope of our systematic work, we became inter­ested in pyridine-4-carbo­thio­amide as another ligand that was reacted with cadmium(II) thio­cyanate to give the title compound.
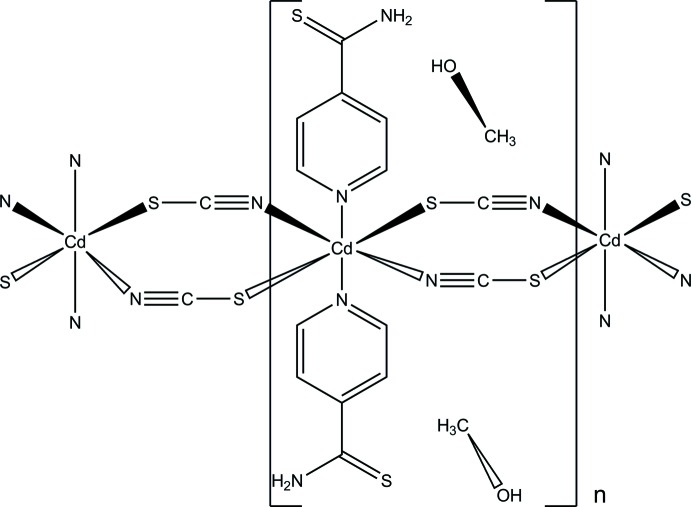


## Structural commentary   

**Figure 1:**
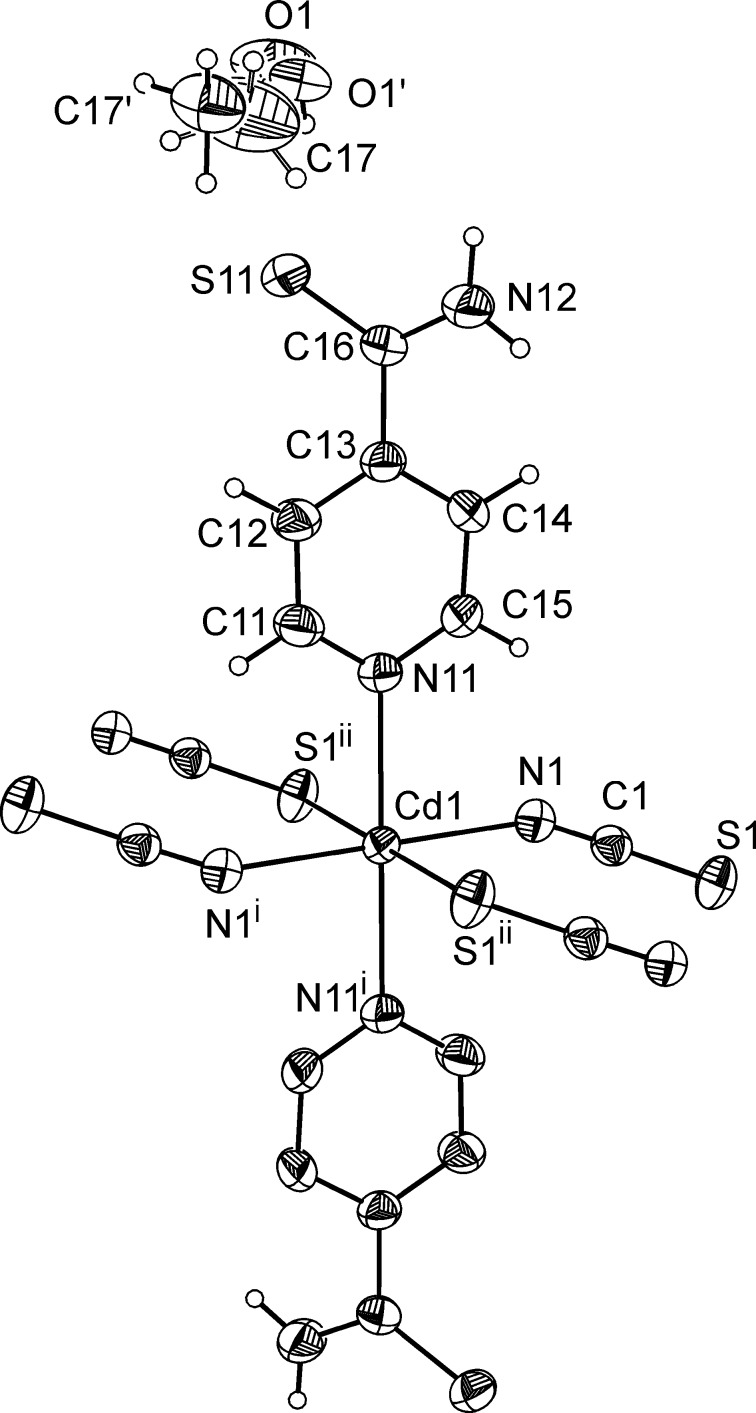
The coordination of the Cd^II^ cation in the structure of the title compound; the two orientations of the methanol solvent mol­ecule are shown. Displacement ellipsoids are drawn at the 50% probability level. [Symmetry codes: (i) −*x* + 1, −*y* + 1, −*z* + 1; (ii) *x*, *y* + 1, *z*; (iii) −*x* + 1, −*y* + 2, −*z* + 1.]

**Figure 2:**
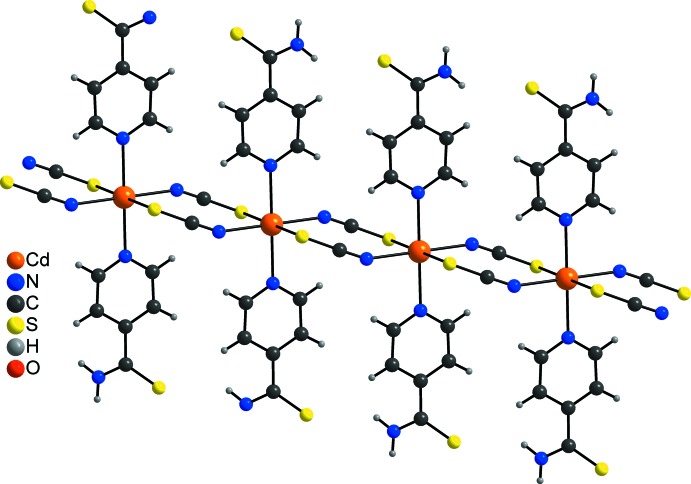
View of a Cd–thio­cyanate chain in the crystal structure of the title compound.

The asymmetric unit of the title compound, [Cd(NCS)_2_(C_6_H_6_N_2_S)]·2CH_3_OH, consists of a Cd^II^ cation that is located on a centre of inversion as well as one thio­cyanato anion, one pyridine-4-carbo­thio­amide ligand and one methanol mol­ecule in general positions. The Cd^II^ cation is sixfold coordinated by two N-bonding pyridine­thio­amide ligands as well as two N- and two S-coordinating thio­cyanate anions in an all *trans* distorted octa­hedral environment (Fig. 1 ▸). As expected, the Cd—N bond length to the negatively charged thio­cyanate anion is significantly shorter than to the pyridine-4-carbo­thio­amide N atom; the Cd—S bond length is within the normal range (Table 1 ▸). The Cd^II^ cations are linked by centrosymmetric pairs of anionic ligands into chains along [010] (Fig. 2 ▸). The methanol mol­ecule is equally disordered over two orientations.

## Supra­molecular features   

**Figure 3:**
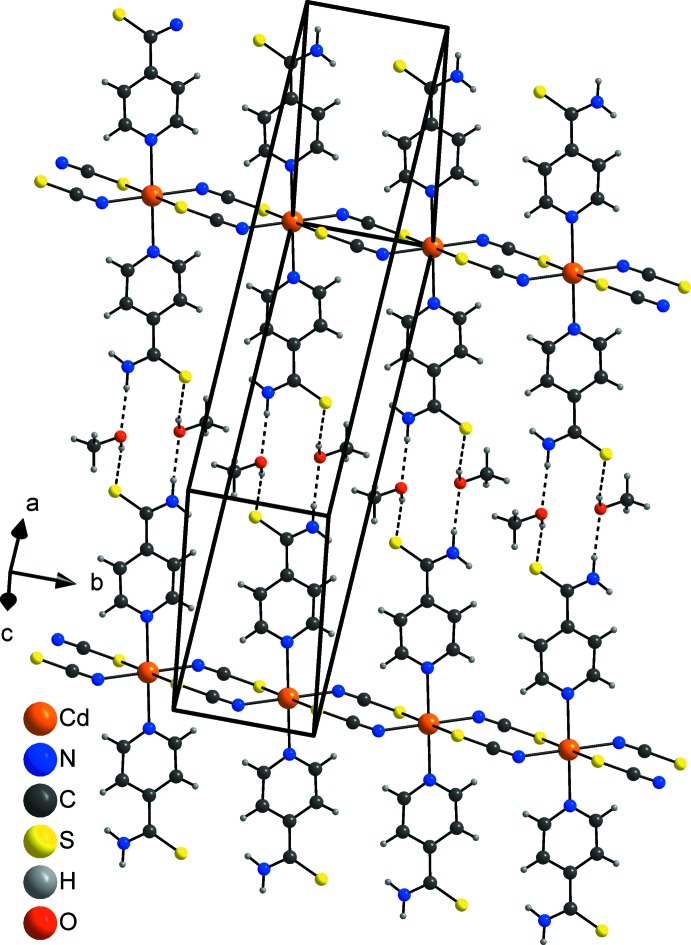
View of one layer in the crystal structure of the title compound with hydrogen bonds shown as dashed lines. Only one orientation of the disordered methanol mol­ecule is shown.

In the crystal structure, the neutral chains are linked into layers extending along (201) by inter­molecular N—H⋯O and O—H⋯S hydrogen bonding *via* the methanol solvent mol­ecules (Fig. 3 ▸). Each pyridine-4-carbo­thio­amide ligand of neighbouring chains makes one N—H⋯O hydrogen bond to the hydroxyl O atom that acts as an acceptor, and one O—H⋯S hydrogen bond between the hydroxyl H atom and the S atom of the pyridine-4-carbo­thio­amide ligand (Fig. 3 ▸ and Table 2 ▸). The hydrogen-bonding geometry is very similar for the two disordered and slightly differently oriented methanol mol­ecules (Table 2 ▸). This arrangement leads to 12-membered rings [graph-set notation 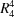
(12); Etter *et al.*, 1990 ▸] in which four donor and four acceptors are involved (Fig. 2 ▸ and Table 2 ▸). There are additional C—H⋯N, C—H⋯S and N—H⋯S inter­actions of much weaker nature that consolidate the three-dimensional network (Table 2 ▸).

## Database survey   

According to the Cambridge Structural Database (Version 5.36, last update 2015; Groom & Allen, 2014 ▸) no coordination compounds with pyridine-4-carbo­thio­amide have been structurally characterized. There is only one crystal structure of the ligand itself reported at room temperature and at 100 K (Colleter & Gadret, 1967 ▸; Eccles *et al.*, 2014 ▸). The crystal structure of the protonated ligand 4-thio­carbamoylpyridinium iodide was also reported recently (Shotonwa & Boeré, 2014 ▸).

## Synthesis and crystallization   

CdSO_4_·3/8H_2_O was purchased from Merck and pyridine-4-carbo­thio­amide and Ba(NCS)_2_·3H_2_O were purchased from Alfa Aesar. Cd(NCS)_2_ was synthesized by stirring 17.5 g (57.0 mmol) Ba(NCS)_2_·3H_2_O and 14.6 g (57.0 mmol) CdSO_4_·3/8H_2_O in 300 ml water at room temperature for 3 h. The white residue of BaSO_4_ was filtered off and the resulting solution dried at 353 K. The homogeneity of the product was checked by X-ray powder diffraction and elemental analysis. The title compound was obtained by reaction of 11.4 mg Cd(NCS)_2_ (0.05 mmol) with 27.6 mg pyridine-4-carbo­thio­amide (0.2 mmol) in boiling methanol (2 ml). Crystals suitable for single-crystal x-ray diffraction formed after cooling.

## Refinement details   

Crystal data, data collection and structure refinement details are summarized in Table 3 ▸. The C—H, O—H and N—H hydrogen atoms were located in a difference map but were positioned with idealized geometry (methyl and O—H hydrogen atoms allowed to rotate but not to tip) and were refined with *U*_iso_(H) = 1.2*U*_eq_(C, N) (1.5 for methyl and O—H hydrogen atoms) using a riding model with C—H = 0.95 Å for aromatic, C—H = 0.98 Å for methyl, N—H = 0.88 Å and O—H = 0.84 Å, respectively. The methanol mol­ecule is equally disordered over two orientations and was refined using a split model using SAME restraints (Sheldrick, 2015 ▸).

## Supplementary Material

Crystal structure: contains datablock(s) I, New_Global_Publ_Block. DOI: 10.1107/S2056989016002632/wm5271sup1.cif

Structure factors: contains datablock(s) I. DOI: 10.1107/S2056989016002632/wm5271Isup2.hkl

CCDC reference: 1453442

Additional supporting information:  crystallographic information; 3D view; checkCIF report

## Figures and Tables

**Table 1:** Selected bond lengths (Å)

Cd1—N1	2.3212 (18)	Cd1—S1^i^	2.7174 (6)
Cd1—N11	2.3576 (18)		

Symmetry code: (i) 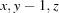.

**Table 2:** Hydrogen-bond geometry (Å, °)

*D*—H⋯*A*	*D*—H	H⋯*A*	*D*⋯*A*	*D*—H⋯*A*
C11—H11⋯N1^ii^	0.95	2.69	3.335 (3)	126
C12—H12⋯S1^iii^	0.95	2.86	3.762 (2)	158
C15—H15⋯N1	0.95	2.54	3.230 (3)	130
N12—H12*B*⋯O1^iv^	0.88	2.02	2.883 (10)	165
N12—H12*B*⋯O1′^iv^	0.88	1.88	2.740 (12)	165
N12—H12*A*⋯S1^v^	0.88	2.90	3.560 (3)	133
N12—H12*A*⋯S11^vi^	0.88	2.87	3.522 (2)	132
O1—H1⋯S11	0.84	2.53	3.351 (12)	165
O1′—H1′⋯S11	0.84	2.46	3.250 (12)	156

Symmetry codes: (ii) 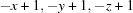; (iii) 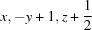; (iv) 
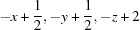
; (v) 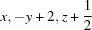; (vi) 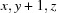.

**Table 3:** Experimental details

Crystal data	
Chemical formula	[Cd(NCS)_2_(C_6_H_6_N_2_S)]·2CH_4_O
*M*_r_	1138.04
Crystal system, space group	Monoclinic, *C*2/*c*
Temperature (K)	200
*a*, *b*, *c* (Å)	25.1891 (10), 5.8729 (2), 15.5080 (6)
β (°)	90.124 (3)
*V* (Å^3^)	2294.14 (15)
*Z*	2
Radiation type	Mo *K*α
μ (mm^−1^)	1.34
Crystal size (mm)	0.18 × 0.14 × 0.10

Data collection	
Diffractometer	Stoe *IPDS2*
Absorption correction	Numerical (*X-SHAPE* and *X-RED32*; Stoe, 2008 ▸)
*T*_min_, *T*_max_	0.644, 0.800
No. of measured, independent and observed [*I* > 2σ(*I*)] reflections	15896, 2515, 2208
*R*_int_	0.032
(sin θ/λ)_max_ (Å^−1^)	0.639

Refinement	
*R*[*F* ^2^ > 2σ(*F* ^2^)], *wR*(*F* ^2^), *S*	0.026, 0.067, 1.06
No. of reflections	2515
No. of parameters	156
No. of restraints	37
H-atom treatment	H-atom parameters constrained
Δρ_max_, Δρ_min_ (e Å^−3^)	0.41, −0.47

Computer programs: *X-AREA* (Stoe, 2008 ▸), *SHELXS97* and *XP* in *SHELXTL* (Sheldrick, 2008 ▸), *SHELXL2014* (Sheldrick, 2015 ▸), *DIAMOND* (Brandenburg, 1999 ▸) and *publCIF* (Westrip, 2010 ▸).

## supplementary crystallographic information

### Crystal data

**Table:**

[Cd(NCS)_2_(C_6_H_6_N_2_S)]·2CH_4_O	*F*(000) = 1144
*M**_r_* = 1138.04	*D*_x_ = 1.647 Mg m^−^^3^
Monoclinic, *C*2/*c*	Mo *K*α radiation, λ = 0.71073 Å
*a* = 25.1891 (10) Å	Cell parameters from 16834 reflections
*b* = 5.8729 (2) Å	θ = 1.3–27°
*c* = 15.5080 (6) Å	µ = 1.34 mm^−^^1^
β = 90.124 (3)°	*T* = 200 K
*V* = 2294.14 (15) Å^3^	Block, colorless
*Z* = 2	0.18 × 0.14 × 0.10 mm

### Data collection

**Table:**

Stoe IPDS-2 diffractometer	2208 reflections with *I* > 2σ(*I*)
ω scans	*R*_int_ = 0.032
Absorption correction: numerical (*X-SHAPE* and *X-RED32*; Stoe, 2008)	θ_max_ = 27.0°, θ_min_ = 1.6°
*T*_min_ = 0.644, *T*_max_ = 0.800	*h* = −32→32
15896 measured reflections	*k* = −7→7
2515 independent reflections	*l* = −19→19

### Refinement

**Table:**

Refinement on *F*^2^	Hydrogen site location: inferred from neighbouring sites
Least-squares matrix: full	H-atom parameters constrained
*R*[*F*^2^ > 2σ(*F*^2^)] = 0.026	*w* = 1/[σ^2^(*F*_o_^2^) + (0.0471*P*)^2^] where *P* = (*F*_o_^2^ + 2*F*_c_^2^)/3
*wR*(*F*^2^) = 0.067	(Δ/σ)_max_ < 0.001
*S* = 1.06	Δρ_max_ = 0.41 e Å^−^^3^
2515 reflections	Δρ_min_ = −0.47 e Å^−^^3^
156 parameters	Extinction correction: *SHELXL2014* (Sheldrick, 2015), Fc^*^=kFc[1+0.001xFc^2^λ^3^/sin(2θ)]^-1/4^
37 restraints	Extinction coefficient: 0.0015 (2)

### Special details

**Table:**

Geometry. All e.s.d.'s (except the e.s.d. in the dihedral angle between two l.s. planes) are estimated using the full covariance matrix. The cell e.s.d.'s are taken into account individually in the estimation of e.s.d.'s in distances, angles and torsion angles; correlations between e.s.d.'s in cell parameters are only used when they are defined by crystal symmetry. An approximate (isotropic) treatment of cell e.s.d.'s is used for estimating e.s.d.'s involving l.s. planes.

### Fractional atomic coordinates and isotropic or equivalent isotropic displacement parameters (Å^2^)

**Table:**

	*x*	*y*	*z*	*U*_iso_*/*U*_eq_	Occ. (<1)
Cd1	0.5000	0.5000	0.5000	0.02964 (10)	
N1	0.45141 (7)	0.8201 (3)	0.46141 (12)	0.0363 (4)	
C1	0.44617 (9)	1.0011 (3)	0.43255 (13)	0.0317 (4)	
S1	0.43930 (2)	1.25424 (10)	0.38878 (4)	0.04069 (15)	
N11	0.43968 (8)	0.4286 (3)	0.61284 (12)	0.0361 (4)	
C11	0.44093 (9)	0.2407 (4)	0.66130 (15)	0.0426 (5)	
H11	0.4684	0.1332	0.6514	0.051*	
C12	0.40419 (9)	0.1946 (4)	0.72524 (14)	0.0405 (5)	
H12	0.4060	0.0563	0.7569	0.049*	
C13	0.36515 (9)	0.3512 (4)	0.74238 (13)	0.0353 (5)	
C14	0.36407 (12)	0.5485 (4)	0.6943 (2)	0.0542 (7)	
H14	0.3381	0.6623	0.7050	0.065*	
C15	0.40177 (12)	0.5776 (5)	0.62990 (18)	0.0532 (7)	
H15	0.4002	0.7124	0.5962	0.064*	
C16	0.32407 (9)	0.3067 (4)	0.81005 (14)	0.0366 (5)	
N12	0.31373 (11)	0.4768 (4)	0.86169 (16)	0.0540 (6)	
H12A	0.3305	0.6070	0.8554	0.065*	
H12B	0.2900	0.4610	0.9028	0.065*	
S11	0.29342 (3)	0.05620 (11)	0.81316 (4)	0.04527 (16)	
O1	0.2777 (4)	0.0596 (16)	1.0276 (8)	0.067 (3)	0.5
H1	0.2760	0.0718	0.9737	0.100*	0.5
C17	0.3143 (5)	−0.101 (3)	1.0489 (13)	0.062 (3)	0.5
H17A	0.3476	−0.0703	1.0185	0.093*	0.5
H17B	0.3010	−0.2520	1.0324	0.093*	0.5
H17C	0.3206	−0.0978	1.1113	0.093*	0.5
O1'	0.2639 (4)	−0.0102 (19)	1.0155 (8)	0.079 (3)	0.5
H1'	0.2637	0.0358	0.9642	0.118*	0.5
C17'	0.3144 (8)	−0.019 (4)	1.0447 (17)	0.113 (8)	0.5
H17D	0.3348	0.1072	1.0201	0.169*	0.5
H17E	0.3306	−0.1640	1.0274	0.169*	0.5
H17F	0.3146	−0.0074	1.1077	0.169*	0.5

### Atomic displacement parameters (Å^2^)

**Table:**

	*U*^11^	*U*^22^	*U*^33^	*U*^12^	*U*^13^	*U*^23^
Cd1	0.03369 (13)	0.02444 (13)	0.03081 (13)	−0.00052 (8)	0.00757 (8)	0.00137 (8)
N1	0.0362 (9)	0.0302 (10)	0.0427 (10)	0.0018 (7)	0.0035 (8)	0.0034 (8)
C1	0.0325 (10)	0.0329 (11)	0.0298 (9)	−0.0004 (8)	0.0016 (8)	−0.0038 (8)
S1	0.0504 (3)	0.0295 (3)	0.0421 (3)	−0.0022 (2)	−0.0116 (2)	0.0059 (2)
N11	0.0383 (10)	0.0333 (9)	0.0366 (9)	−0.0026 (8)	0.0111 (8)	0.0000 (8)
C11	0.0392 (12)	0.0483 (13)	0.0404 (11)	0.0086 (10)	0.0107 (9)	0.0094 (10)
C12	0.0404 (12)	0.0444 (13)	0.0368 (11)	0.0058 (10)	0.0090 (9)	0.0105 (10)
C13	0.0377 (11)	0.0342 (11)	0.0341 (10)	−0.0052 (9)	0.0090 (9)	−0.0034 (8)
C14	0.0611 (16)	0.0340 (12)	0.0677 (17)	0.0117 (11)	0.0367 (14)	0.0063 (12)
C15	0.0667 (17)	0.0303 (11)	0.0628 (16)	0.0054 (12)	0.0336 (14)	0.0104 (11)
C16	0.0364 (11)	0.0380 (11)	0.0353 (10)	0.0017 (9)	0.0096 (9)	0.0005 (9)
N12	0.0645 (15)	0.0432 (12)	0.0543 (13)	−0.0078 (10)	0.0297 (11)	−0.0077 (10)
S11	0.0490 (3)	0.0376 (3)	0.0493 (3)	−0.0070 (3)	0.0158 (3)	0.0009 (3)
O1	0.066 (6)	0.063 (4)	0.072 (6)	0.006 (3)	0.043 (5)	0.005 (3)
C17	0.045 (5)	0.091 (7)	0.050 (5)	0.008 (4)	−0.004 (3)	0.005 (5)
O1'	0.059 (5)	0.127 (10)	0.050 (3)	0.006 (5)	0.019 (3)	0.019 (5)
C17'	0.084 (10)	0.18 (2)	0.077 (10)	0.001 (11)	0.003 (7)	−0.013 (12)

### Geometric parameters (Å, º)

**Table:**

Cd1—N1^i^	2.3212 (18)	C14—C15	1.390 (3)
Cd1—N1	2.3212 (18)	C14—H14	0.9500
Cd1—N11^i^	2.3575 (18)	C15—H15	0.9500
Cd1—N11	2.3576 (18)	C16—N12	1.307 (3)
Cd1—S1^ii^	2.7174 (6)	C16—S11	1.662 (2)
Cd1—S1^iii^	2.7174 (6)	N12—H12A	0.8800
N1—C1	1.161 (3)	N12—H12B	0.8800
C1—S1	1.643 (2)	O1—C17	1.361 (13)
S1—Cd1^iv^	2.7174 (6)	O1—H1	0.8400
N11—C15	1.322 (3)	C17—H17A	0.9800
N11—C11	1.335 (3)	C17—H17B	0.9800
C11—C12	1.385 (3)	C17—H17C	0.9800
C11—H11	0.9500	O1'—C17'	1.351 (15)
C12—C13	1.373 (3)	O1'—H1'	0.8400
C12—H12	0.9500	C17'—H17D	0.9800
C13—C14	1.378 (3)	C17'—H17E	0.9800
C13—C16	1.498 (3)	C17'—H17F	0.9800
			
N1^i^—Cd1—N1	180.00 (9)	C12—C13—C16	121.0 (2)
N1^i^—Cd1—N11^i^	89.72 (7)	C14—C13—C16	120.8 (2)
N1—Cd1—N11^i^	90.28 (7)	C13—C14—C15	118.6 (2)
N1^i^—Cd1—N11	90.28 (7)	C13—C14—H14	120.7
N1—Cd1—N11	89.72 (7)	C15—C14—H14	120.7
N11^i^—Cd1—N11	180.0	N11—C15—C14	123.9 (2)
N1^i^—Cd1—S1^ii^	91.67 (5)	N11—C15—H15	118.1
N1—Cd1—S1^ii^	88.33 (5)	C14—C15—H15	118.1
N11^i^—Cd1—S1^ii^	89.20 (5)	N12—C16—C13	115.8 (2)
N11—Cd1—S1^ii^	90.80 (5)	N12—C16—S11	124.43 (17)
N1^i^—Cd1—S1^iii^	88.33 (5)	C13—C16—S11	119.74 (16)
N1—Cd1—S1^iii^	91.67 (5)	C16—N12—H12A	120.0
N11^i^—Cd1—S1^iii^	90.80 (5)	C16—N12—H12B	120.0
N11—Cd1—S1^iii^	89.20 (5)	H12A—N12—H12B	120.0
S1^ii^—Cd1—S1^iii^	180.0	C17—O1—H1	109.5
C1—N1—Cd1	154.23 (17)	O1—C17—H17A	109.5
N1—C1—S1	178.2 (2)	O1—C17—H17B	109.5
C1—S1—Cd1^iv^	99.19 (7)	H17A—C17—H17B	109.5
C15—N11—C11	116.8 (2)	O1—C17—H17C	109.5
C15—N11—Cd1	119.86 (16)	H17A—C17—H17C	109.5
C11—N11—Cd1	123.37 (15)	H17B—C17—H17C	109.5
N11—C11—C12	123.3 (2)	C17'—O1'—H1'	109.5
N11—C11—H11	118.3	O1'—C17'—H17D	109.5
C12—C11—H11	118.3	O1'—C17'—H17E	109.5
C13—C12—C11	119.2 (2)	H17D—C17'—H17E	109.5
C13—C12—H12	120.4	O1'—C17'—H17F	109.5
C11—C12—H12	120.4	H17D—C17'—H17F	109.5
C12—C13—C14	118.2 (2)	H17E—C17'—H17F	109.5

Symmetry codes: (i) −*x*+1, −*y*+1, −*z*+1; (ii) *x*, *y*−1, *z*; (iii) −*x*+1, −*y*+2, −*z*+1; (iv) *x*, *y*+1, *z*.

### Hydrogen-bond geometry (Å, º)

**Table:**

*D*—H···*A*	*D*—H	H···*A*	*D*···*A*	*D*—H···*A*
C11—H11···N1^i^	0.95	2.69	3.335 (3)	126
C12—H12···S1^v^	0.95	2.86	3.762 (2)	158
C15—H15···N1	0.95	2.54	3.230 (3)	130
N12—H12*B*···O1^vi^	0.88	2.02	2.883 (10)	165
N12—H12*B*···O1′^vi^	0.88	1.88	2.740 (12)	165
N12—H12*A*···S1^vii^	0.88	2.90	3.560 (3)	133
N12—H12*A*···S11^iv^	0.88	2.87	3.522 (2)	132
O1—H1···S11	0.84	2.53	3.351 (12)	165
O1′—H1′···S11	0.84	2.46	3.250 (12)	156

Symmetry codes: (i) −*x*+1, −*y*+1, −*z*+1; (iv) *x*, *y*+1, *z*; (v) *x*, −*y*+1, *z*+1/2; (vi) −*x*+1/2, −*y*+1/2, −*z*+2; (vii) *x*, −*y*+2, *z*+1/2.
